# Combined In Vivo Microdialysis and PET Studies to Validate [^11^C]Yohimbine Binding as a Marker of Noradrenaline Release

**DOI:** 10.3390/biom13040674

**Published:** 2023-04-14

**Authors:** Anne Marlene Landau, Steen Jakobsen, Majken Borup Thomsen, Aage Kristian Olsen Alstrup, Dariusz Orlowski, Jan Jacobsen, Gregers Wegener, Arne Mørk, Jens Christian Hedemann Sørensen, Doris J. Doudet

**Affiliations:** 1 Translational Neuropsychiatry Unit, Department of Clinical Medicine, Aarhus University, A701, Palle Juul Jensens Boulevard 99, 8200 Aarhus, Denmark; 2 Department of Nuclear Medicine & PET-Center, Aarhus University Hospital, 8200 Aarhus, Denmark; 3 Center for Experimental Neuroscience (CENSE), Department of Neurosurgery, Aarhus University Hospital, 8200 Aarhus, Denmark; 4 Synaptic Transmission, H. Lundbeck A/S, Ottiliavej 9, Valby, 2500 Copenhagen, Denmark; 5 Department of Medicine/Neurology, University of British Columbia, 2221 Wesbrook Mall, Vancouver, BC V6T 2B5, Canada; ddoudet@mail.ubc.ca

**Keywords:** a2 adrenoceptors, noradrenaline, microdialysis, minipig, positron emission tomography, [^11^C]yohimbine

## Abstract

The noradrenaline system attracts attention for its role in mood disorders and neurodegenerative diseases but the lack of well-validated methods impairs our understanding when assessing its function and release in vivo. This study combines simultaneous positron emission tomography (PET) and microdialysis to explore if [^11^C]yohimbine, a selective antagonist radioligand of the α2 adrenoceptors, may be used to assess in vivo changes in synaptic noradrenaline during acute pharmacological challenges. Anesthetised Göttingen minipigs were positioned in a head holder in a PET/CT device. Microdialysis probes were placed in the thalamus, striatum and cortex and dialysis samples were collected every 10 min. Three 90 min [^11^C]yohimbine scans were acquired: at baseline and at two timepoints after the administration of amphetamine (1–10 mg/kg), a non-specific releaser of dopamine and noradrenaline, or nisoxetine (1 mg/kg), a specific noradrenaline transporter inhibitor. [^11^C]yohimbine volumes of distribution (V_T_) were obtained using the Logan kinetic model. Both challenges induced a significant decrease in yohimbine V_T_, with time courses reflecting their different mechanisms of action. Dialysis samples revealed a significant increase in noradrenaline extracellular concentrations after challenge and an inverse correlation with changes in yohimbine V_T_. These data suggest that [^11^C]yohimbine can be used to evaluate acute variations in synaptic noradrenaline concentrations after pharmacological challenges.

## 1. Introduction

Altered noradrenaline (NA) neurotransmission is implicated in neurodegenerative diseases such as Parkinson’s and Alzheimer’s diseases, as well as in psychiatric conditions, including mood disorders and attention deficit hyperactivity disorder [1,2]. However, the exact role and mechanism of action of NA in disease pathogenesis and associated treatments is unclear, impeded until recently by the lack of adequate selective in vivo markers.

Understanding the role of dopamine (DA) in brain function has been facilitated by the availability of selective ligands for their pre- and post-synaptic function. However, the major breakthrough in understanding its functional significance was in the discovery of ligands that could be used as surrogate markers of acute DA release. Specifically, the discovery that benzamides such as IBZM, CITs or raclopride, single photon emission computed tomography (SPECT) and PET antagonists of the D2/3 receptors, can be used to image DA release [3,4,5] opened a new era of study with a shift in focus from regional receptor distribution and density to the importance of subtle variations in the synaptic environment in response to pharmacological or psychological challenges [6,7,8]. This has led to understanding the role of alterations in functional DA levels in response to events between individuals and populations [5,8,9] whether or not the number of receptors at baseline is affected. There is thus a need to develop better tools to similarly assess the role of NA in live subjects.

Our team has developed [^11^C]yohimbine, an antagonist of the α2-adrenergic NA receptors, as a tracer for PET brain imaging studies [10]. Although [^11^C]yohimbine is not fully specific for α2 receptors and, at pharmacological doses, has some activity at the α1 and 5HT1A sites, in tracer concentrations, it exhibits a high selectivity for all of the α2 receptor binding sites in the living brain. The baseline distribution of [^11^C]yohimbine has regional differences consistent with the known in vitro distribution of α2 receptors, with cortex and thalamus > mesencephalon > cerebellum, pons and medulla [11,12,13]. Central α2-adrenergic receptors are expressed in NA neurons both pre-synaptically in the locus coeruleus (LC), where they control the inhibition of neurotransmitter release from presynaptic nerves, and pre and mostly post-synaptically in the cortex, where they can modulate cellular signalling pathways [14].

Previously, we have used [^11^C]yohimbine PET in Göttingen minipigs to study the acute effects of vagal nerve stimulation (VNS) on noradrenergic neurotransmission [15]. We tested the hypothesis that at least some of the therapeutic effects of brain stimulation in epilepsy and depression were mediated through the restoration of normal cortical inhibition by an increased activity of the NA system through stimulation of the LC through the vagus nerve pontine connections [16]. Indeed, we found a decreased [^11^C]yohimbine binding following VNS [15]. VNS in rodents induces a rapid increase in basal and burst firing in LC NA neurons [17,18,19] and activates noradrenergic modulatory systems [20]. A microdialysis study in rat revealed that VNS at 1.0 mA increased NA release in the cortex and hippocampus [21]. This led to our hypothesis that [^11^C]yohimbine may be a useful surrogate marker of NA release in vivo.

This study aimed to validate the use of [^11^C]yohimbine as a specific surrogate marker of changes in NA release. We have previously shown that the administration of amphetamine, a non-specific releaser of NA and DA, to rat [22] and pig [23] reduces [^11^C]yohimbine binding. Here, we combined PET with simultaneous microdialysis during challenges with two compounds known to increase extracellular NA with different time frames and mechanisms of action. In the first group of animals, we performed challenges with 1, 2 and 10 mg/kg amphetamine intravenously (IV) to induce a dose-dependent release of NA (and DA) and a rapid increase in extracellular NA. A second group of animals received 1 mg/kg of nisoxetine, a selective blocker of the NA transporter, which thus induces a slow increase in extracellular NA over a period of hours. This design helped us to assess in real-time whether changes in [^11^C]yohimbine binding reflect changes in extracellular NA.

## 2. Materials and Methods

### 2.1. Animals

Twelve young adult female Göttingen minipigs weighing 24–36 kg (Ellegaard Minipigs ApS, Dalmose, Zealand, Denmark) were used in accordance with a protocol approved by the Danish Animal Experimentation Inspectorate and were reported in compliance with the ARRIVE guidelines. The size of the minipig brain is adequate for imaging and stereotaxic catheter placement, and the existence of an MRI-based atlas [24] allows for the co-registration of PET data for accurate analysis of the regional distribution of radioligands. Minipigs were fed a restricted pellet diet (SDS Diet, Witham, UK). They were fasted overnight, with free access to tap water, prior to the day of the experiment. Environmental conditions in the animal facility were 20 °C and 50–55% relative humidity, 12:12 h light/dark cycle, and the air was changed 8 times every hour. Pigs were double-housed in a 4.6 m^2^ enclosure with fence-line visual contact with congeners, and environmental enrichment was used. The minipigs were all acclimatised for at least 3 weeks prior to any procedure.

### 2.2. Experimental Setup

The minipigs were randomly assigned to the treatment groups. The minipigs were sedated with a mixture of 1.25 mg/kg midazolam and 6.25 mg/kg s-ketamine intramuscularly (IM) for transport to the PET suite. Upon arrival, an ear vein catheter (21 G Venflon) was inserted and used to induce anaesthesia with a mixture of 1.25 mg/kg midazolam and 3.13 mg/kg s-ketamine IV. The animals were then intubated and anaesthesia was maintained with 2.0–2.3% isoflurane. The minipigs were mechanically ventilated with approximately 8 mL/kg/minute of a mixture of 1 part O_2_ and 2.2 parts medical air. Pulse, arterial oxygen (SaO_2_) and body temperature were monitored throughout the day and maintained at normal values for minipig [25]. Hydration was maintained with a saline drip.

**Figure 1:**
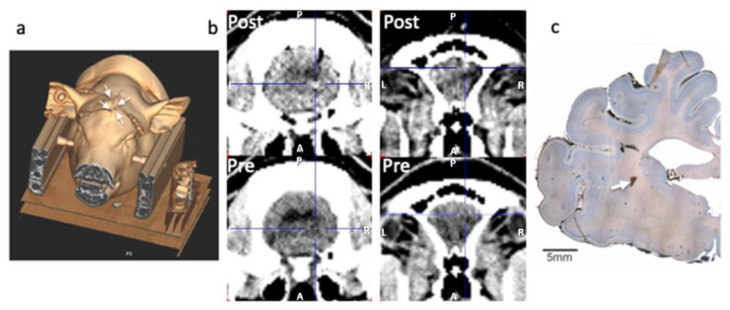
Examples of methods used for validating the animal procedure and location of the microdialysis probes. Three-dimensional reconstruction of the animal in the head holder in the PET/CT gantry, obtained from the CT scan used for attenuation correction (**a**). The head of the animal is securely fixed in the stereotaxic headholder and the animal is advanced in the scanner in a prone position. The microdialysis probes can be seen exiting the closed midline incision (four white arrows) for connection to the pump and collecting vials on ice placed outside of the gantry for easy collection of the samples every 10 min. Two examples of probe location are shown in (**b**): the bottom row shows pre-implant CTs at the level of thalamus and frontal cortex. The top row shows the corresponding post-implant-pre-PET CTs with the gold tip target indicated by the cross hair as a white dot in the top image. An example of poor striatal location of the gold tip of the dialysis probe is shown in (**c**). In this image, the tip location (white arrow) appears to be in the internal capsule, between caudate and putamen. This image, covering an entire hemisphere of the pig brain, is a combination of the 40–50 microphotographs merged in Photoshop software.

Minipigs were placed in a 3-point fixation head-holder designed for compatibility with PET and MRI [26], with two titanium tipped screws in the zygoma bone and a hard palate holder. Analgesia (1 mL bupivacaine 1%) was injected locally at the level of the zygoma screws and 8 mL was injected as infiltration analgesia at the surgical site. Prior to surgical intervention, buprenorphine (1 mL/3 micrograms) was administered IM. A midline incision was performed on top of the head, where local analgesia was applied, and the skull was exposed and dried before two small holes were drilled at the level of bregma and lambda to permit insertion of two temporary fiducial markers [27]. A high-resolution transmission CT scan (500 mAS, 120 kV, 1.0 mm 64 × 0.6 mm slice, 0.8 pitch) was obtained that allowed for sufficient identification of the skull, brain structures and fiducial markers in order to determine antero-posterior, lateral and dorso-ventral stereotaxic implant coordinates for the microdialysis probes (see Figure 1). The fiducial marker screws were removed and small holes were drilled above the regions of interest. The dura was gently punctured with a syringe needle tip to permit insertion of the probes into the brain parenchyma (Human CMA70 dialysis probes; membrane length: 1 cm; shaft: 10 cm). The probe shafts were cut to allow the membrane a 1 cm protrusion into the brain parenchyma and their descent to the pre-determined depth and location in striatum, thalamus and cortex, while the shafts had a tight fit in the burr holes and were firmly secured there with a surgical adhesive (Bioglue^®^). The skin was then loosely sutured around the probe shafts. Figure 1a shows a 3D reconstruction of the pig’s head in the scanner after implant of the dialysis probes (exit location of the probes shown by white arrows). This reconstruction was made from the CT scan obtained immediately prior to the PET studies for attenuation connection (50 mAS, 120 kV, 5.0 mm 24 × 1.2 mm slice, 0.8 pitch).

Each probe was connected to a standard microdialysis assembly located outside the gantry of the PET/CT device, including a pump (CMA 400 syringe pump), syringe, tubing and glass vials (Microlab Aarhus A/S, Denmark) containing 5 µL of perchloric acid (5 mM HClO_4_ and 100 µM EDTA), kept on ice for collection of the dialysate. The pump was started after insertion of the probes and perfusion was continued for 2–3 h to allow for equilibration before starting collection of baseline samples. Standard artificial cerebrospinal fluid (CSF) at a flow of 2 μL/min was perfused throughout the entire experiment. Baseline samples were collected for at least 2 h prior to challenge. The baseline yohimbine imaging data were acquired during this baseline collection prior to challenge.

The microdialysis probes were inserted in one striatum and bilaterally in the thalamus. We tentatively tried to implant one probe in the frontal pole with the understanding that the length of the human dialysis probe tip (10 mm) and the shape and thickness (mm) of cortex in this region may increase variability in placement and percentage of white vs. grey matter surrounding the dialysis membrane and thus make measurements subject to high variability. Sampling from each region continued without interruption, every 10 min throughout the day, for at least two hours at baseline (starting 2 h after implant), during and in between scans. Sampling lasted up to 6 h after the drug challenge, including during the 2 post-challenge [^11^C]yohimbine scans, to allow for direct comparison of PET and microdialysis data.

Vital signs (heart rate, respiratory rate, pO_2_, pCO_2_ and temperature) were monitored in the anesthetised pigs throughout the day. The anesthetised minipigs were euthanised by IV administration of a high dose of sodium pentobarbital (100 mg/kg IV). The microdialysis probes were carefully removed and examined for possible damages or blood clots on the dialysis membrane that may indicate unusable data. The brain was removed and coronal sections at the level of the implants were performed to confirm the location of the probes (Figure 1c, white arrow).

### 2.3. Tomography

**Figure 2:**
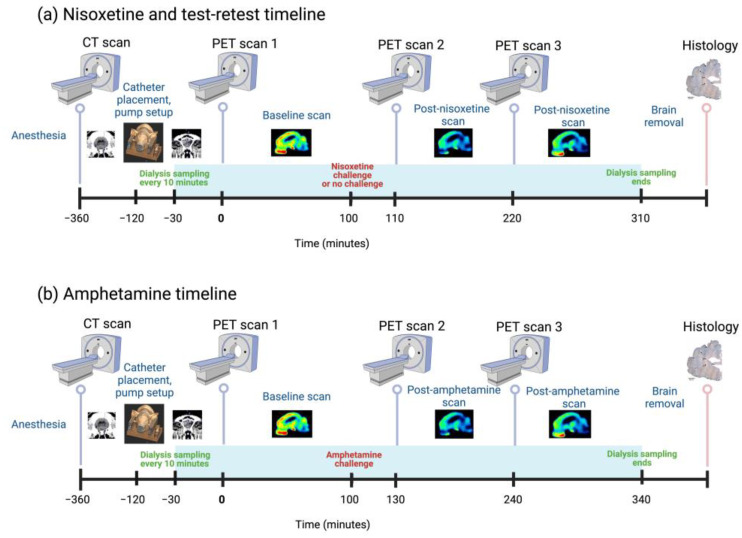
Timeline for experimental studies of drug challenges with either (**a**) 1 mg/kg nisoxetine or (**b**) 1, 2 or 10 mg/kg amphetamine. An overview of the general experimental setup in which minipigs were first deeply anesthetised and CT scanned. Minipigs then underwent surgical catheter placement in frontal cortex, striatum and thalamus, followed by a second CT scan to verify probe placement. We then sampled the dialysate every 10 min, alternated collection for NA and DA analysis and continued until the end of the PET scans. [^11^C]yohimbine PET was performed at baseline (set as time 0) and at two temporal points after each challenge. Please note that the test–retest experiments followed the same timeline as nisoxetine and that the times indicated in each panel are a best approximation of the actual times in each animal. Created with BioRender.com (accessed on 11 April 2023).

[^11^C]yohimbine synthesis has been described previously [10]. Using a Siemens Biograph True point 64 PET/CT (Siemens, Erlangen, Germany), a brief low-energy CT (50 mAS, 120 kV, 5.0 mm 24 × 1.2 mm slice, 0.8 pitch) was obtained for attenuation correction prior to the first PET scan of the day. The baseline scan was acquired 3–4 h following implant of the dialysis probes, allowing for equilibration of the probes and stabilisation of dialysis measurements. Throughout the day, the minipigs received 3 high-specific-activity [^11^C]yohimbine scans with a new batch of tracer synthesized for each injection, and with each scan lasting 90 min. Each tracer dose (200–400 MBq) was adjusted to a 10 mL volume with sterile saline and injected IV over ½–1 min. The mass of yohimbine administered was 0.16–1.8 μg per scan, i.e., well below the mass necessary to induce pharmacological effects. Following the baseline PET scan, a pharmacological challenge was administered IV: 5 animals received R-amphetamine (1 mg/kg (N = 2), 2 mg/kg (N = 2) or 10 mg/kg (N = 1)), a potent NA releaser, and 4 animals received nisoxetine (1 mg/kg), a specific NA transporter (NET) blocker. Three additional animals were used for test–retest studies under the same experimental paradigm. Accounting for delays in synthesis, quality control or synthesis failures, [^11^C]yohimbine scans were started between 15 and 30 min and 185 and 270 min after amphetamine and between 5 and 20 min and 110 and 200 min after nisoxetine. The challenge-control scans (saline or no intervention) were started during the same time frames as the nisoxetine scans. See Figure 2 for a timeline of the experiment.

The PET data were reconstructed using TrueX 3D OSEM (3 iterations, 21 subsets), a 256 × 256 × 109 matrix and a 2 mm Gauss filter and a time-frame structure of 5 × 60, 3 × 300, 4 × 600, and 2 × 900 s (total 14 frames, 90 min). As yohimbine is not metabolised in pigs [10], no arterial input function was obtained.

### 2.4. PET Data Analysis

Data were analysed by a researcher blinded to the treatment groups. The summed frame of the baseline PET data was registered to an MRI atlas of the Göttingen minipig brain [24] using Montreal Neurological Institute tools. Since the animal was not moved in between scans, the same transformation was then applied to the kinetic data of all 3 scans. Before further processing, however, the quality of the registration was checked individually for all 3 scans and adjusted if needed. Time activity curves for striatum, thalamus, occipital, frontal and parietal cortices were generated based on volumes of interest from the MRI atlas of the Göttingen minipig brain [24]. The total volume of distribution (V_T_) was calculated using the Logan graphical analysis using a validated population plasma activity curve corrected for injected dose and weight as the input function [15]. PMOD version 3.7 was used to generate the parametric maps (V_T_) of the representative examples of nisoxetine and amphetamine PET scans.

### 2.5. HPLC Analysis of Dialysates

The samples were divided into 2 sets: the odd-numbered samples were analysed in a high-performance liquid chromatography (HPLC) system optimised explicitly for NA at Lundbeck, Copenhagen. The majority of even-numbered samples were assigned for analysis of DA in the striatum, while samples from the early studies (mostly from the 10 mg amphetamine study) served as practice for set-up and optimisation of the HPLC columns for NA and DA. The content of NA and DA in the microdialysis samples was analysed by means of HPLC with electrochemical detection (ESA Biosciences) as previously described [28,29]. Fifteen µL samples were injected automatically into the HPLC system and monoamines were separated by reverse phase liquid chromatography (MD-150 (C18), 150 × 2 mm (3 µm) column (ESA no. 70-4129). For the NA measurements, the mobile phase consisted of 75 mM lithium acetate, 4 mM sodium 1-heptanesulphonic acid, 100 µM EDTA and 7% methanol (pH 4.7), and the flow rate was 0.17 mL/minute. For the analysis of DA, the mobile phase consisted of 75 mM sodium dihydrogen phosphate monohydrate, 1.7 mM 1-octanesulfonic acid sodium salt, 100µL/L triethylamine, 25 µM EDTA and 10% methanol (pH 3.0), and the flow rate was 0.5 mL/minute. Electrochemical detection for all dialysates was accomplished by using a coulometric analytical cell (ESA 5014B) and cell potential set at E2 = +250 mV (Coulochem II, ESA, Chelmsford, MA, USA). Concentrations of NA and DA were calculated using Chromeleon 6.4 data system (Dionex, Chelmsford, MA, USA).

### 2.6. Statistical Analysis

The PET data were initially analysed regionally for comparison with the microdialysis data. However, as there were no significant differences between the left and right V_T_, or in between the data for the frontal, parietal and occipital cortical regions, in order to reduce variability and the number of comparisons, an average V_T_ of all the cortical regions was used for further comparison and correlations of the effects of the pharmacological challenges. Because of high variability and the small number of animals (N = 3 or 4 for each challenge) in which both the dialysis and PET data survived quality control, we are presenting the data qualitatively only.

Furthermore, in order to analyse the relationship between NA and yohimbine, we considered the known distribution of the α2 subtypes and NA and DA terminals. Cortex and thalamus receive a large (heterogenous in distribution and subtypes) NA input but a sparse DA input and contain mostly α2A and B receptor subtypes [12,13]. The striatum receives relatively few NA terminals and boasts massive DA concentrations. However, it contains a large proportion of α2 receptors, the majority identified as α2C [30], leading to the suggestion that the ligand of the α2C receptors was primarily DA [31]. Thus, since yohimbine does not distinguish between subtypes, for the comparisons between dialysis and scan data, the NA and yohimbine data from cortical and thalamic regions were pooled within individual animals, whereas the striatal data were considered separately.

The microdialysis samples were matched to the scan times and, for comparison with the PET data, the concentrations of NA were expressed as the average concentration during the duration of each scan. This average concentration was then also expressed as the percent change in cortical/thalamic NA from baseline as a result of the drug challenge. For both dialysis and PET, the percent difference between baseline and challenge was calculated as ((post-challenge − baseline)/baseline × 100) for the combined cortical–thalamic data. Correlations between the changes in yohimbine binding and extracellular NA were calculated separately for cortical/thalamic regions and striatum.

### 2.7. Verification of the Probe Location and Histology

The position of the gold thread in the tip of the microdialysis catheter can be visualised on a CT scan. Verification of the probe location in the brain was performed initially in vivo by comparison of the initial high-resolution CT (pre-implant) with the low-resolution CT scan performed immediately prior to the PET scan for attenuation correction of the PET data. The anatomical location was confirmed using pig brain atlases [32,33,34]. An example of pre- and post-CT comparison is shown in Figure 1b.

The probe location was further confirmed in post-mortem brain sections. Briefly, paraformaldehyde-fixed brains were pre-sectioned into 1 cm thick tissue slabs, and slabs containing the probe entrance hole were selected and cryoprotected by immersion in 30% sucrose for 7 days. The tissue blocks were then embedded in Tissue-Tek^®^ OCT and frozen in isopentane cooled with dry ice. Finally, the blocks were sectioned at 40 µm in the coronal plane using a cryomicrotome. To visualise anatomical structures, the brain sections were stained with Toluidine Blue (Nissl), dehydrated with 99% ethanol followed by xylene and coverslipped using Pertex. Colour photographs of the microscopic sections were taken with a Leica DFC 480 camera mounted on a Leica DM5000B microscope using 1.25× objective. The resulting images are a combination of the 80–100 microphotographs merged in Photoshop software (version 24.3).

Upon removal of the probes from the brain, clotted blood was noticed on the tip of two of the probes and the data from these probes were negative throughout the sampling day. The tip of 3 more probes appeared slightly bent/cracked, suggesting damage at insertion or during removal from the brain. If the HPLC data suggested that the probes were damaged at insertion (poor recovery or no measurement), data from these probes were excluded. The entire datasets from 2 animals (1 nisoxetine, 1 low dose amphetamine) were lost due to an unnoticed freezer failure. The data from 2 cortical probes were excluded as we could not confirm the location and, as a whole, the cortical probes provided the most variable data and were located as much in white matter as in grey matter. The tip of one striatal probe was located in white matter (internal capsule) and was excluded (Figure 1c). The other probes were located mostly in cortical or thalamic grey matter (Figure 1b) but the size of the dialysis membrane (10 mm), while permitting a wide sampling area, made it difficult to remain localised completely within one grey matter region, likely increasing sampling variability. We therefore lost the data from a total of 16 probes and considered the results from the remaining 32 probes.

## 3. Results

**Figure 3:**
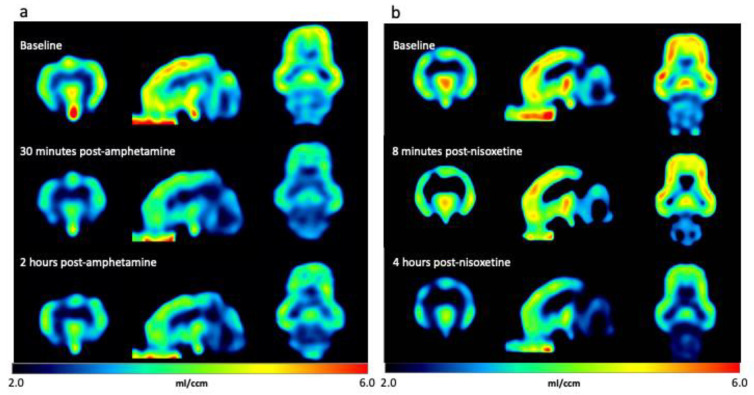
Representative voxel-wise [^11^C]yohimbine volume of distribution parametric maps in minipig brain at baseline and after drug challenges. Data are presented for scans before and after challenges with (**a**) 1 mg/kg amphetamine (baseline (top), 30 min post-challenge (middle) and 2 h post-challenge (bottom)) and (**b**) 1 mg/kg nisoxetine at baseline (top), 8 min post-challenge (middle) and 4 h post-challenge (bottom). Data are shown in coronal (left), sagittal (middle) and axial (right) views. Note that the areas of highest binding at baseline are found in the thalamus, cortex and brainstem, and binding is displaced at different times during the two challenges.

In response to our initial challenge with 10 mg/kg amphetamine, clear decreases in [^11^C]yohimbine V_T_ were observed 30 min post-challenge, which were most prominent in areas of high binding, such as the cortex and thalamus. The decreased V_T_ persisted at the time of the final scan 4 h post-challenge. After the first 10 mg/kg amphetamine scan and validation of our “proof of concept” that we could indeed measure an effect by both PET and dialysis, we decided to reduce the dose of amphetamine administered to be more comparable to those used in human studies while still inducing a robust response. Figure 3 shows the parametric maps of [^11^C]yohimbine V_T_ in minipigs challenged with 1 mg/kg amphetamine and 1 mg/kg nisoxetine. Images are shown for each animal at baseline and at two timepoints after each drug challenge. Both low doses of amphetamine (1 and 2 mg/kg) produced a more moderate but still measurable effect (Figure 3a). Administration of 1 mg/kg nisoxetine resulted in only a slight reduction in V_T_ at the time of the scan 5–20 min post-drug challenge, and decreased dramatically at the scan 2 h post-drug challenge (Figure 3b).

**Figure 4:**
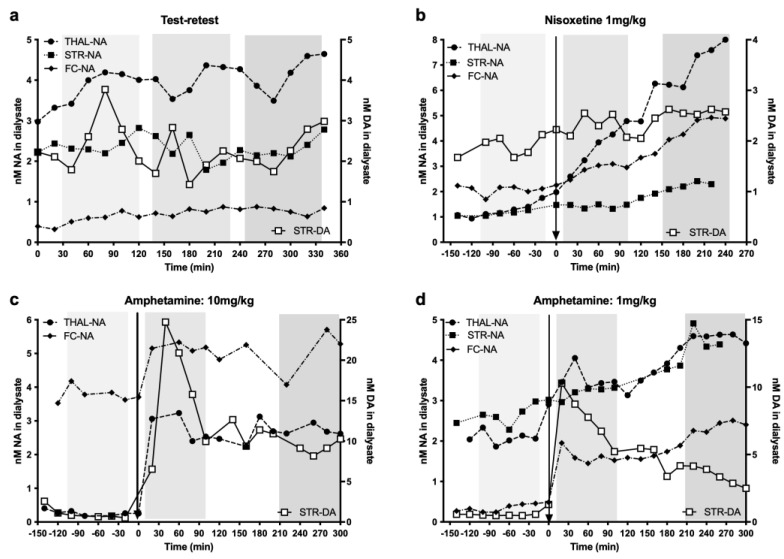
Example of the changes in extracellular NA concentration in response to various challenges: (**a**) saline (test–retest), (**b**) nisoxetine (1 mg/kg) and at 2 doses of amphetamine: (**c**) 10 mg/kg and (**d**) 1 mg/kg. The vertical light-grey-shaded columns indicate the times during which the PET scans were acquired at baseline and after challenge. Note the lack of large, sustained changes throughout the day in the absence of a challenge (test–retest) and the progressive increase in NA after administering nisoxetine. In contrast, note the sharp rise in NA after amphetamine. As an index of specificity, please also note the lack of large changes in striatal DA (open square) in test–retest and nisoxetine (a specific NET inhibitor) and the large increase following amphetamine.

Using the described microdialysis setup, we were able to acquire dialysis data in all 12 pigs; however, as described in Section 2.7 of Methods, the data from 16 probes were lost or excluded. Figure 4 shows a representative example of the raw dialysis data for one animal in each of the challenge groups: (a) test–retest control condition, (b) 1 mg/kg nisoxetine, (c) 10 mg/kg amphetamine and (d) 1 mg/kg amphetamine. The baseline concentrations of NA were variable between animals and between regions, but overall, the changes remained consistent within the pharmacological challenges.

**Figure 5:**
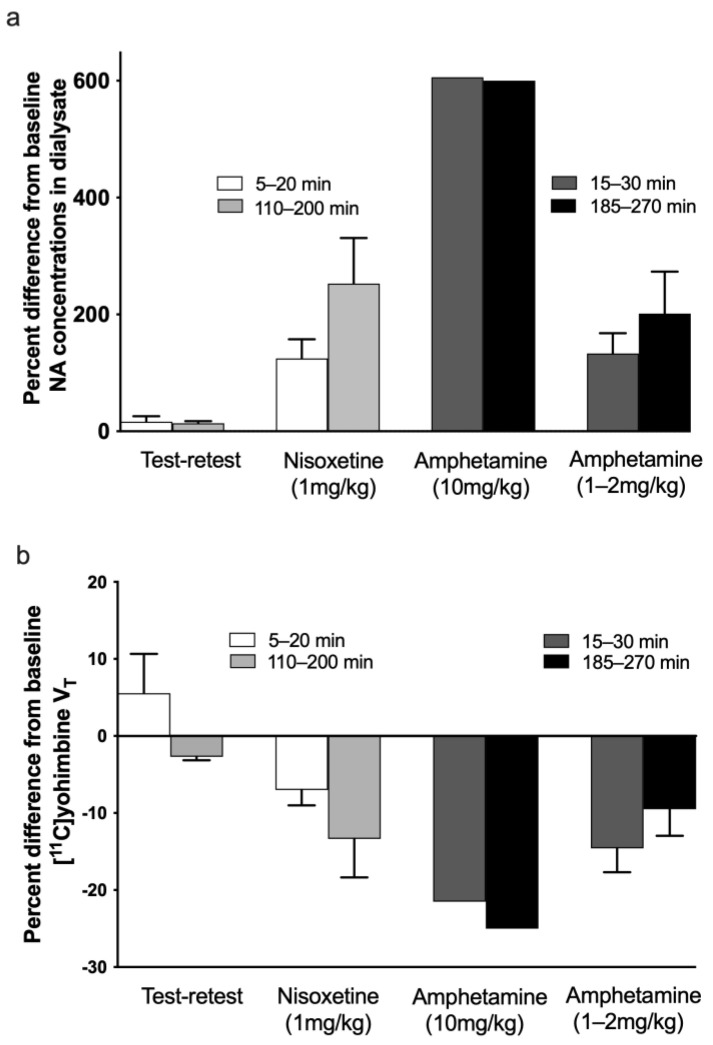
Averaged percent change from baseline to post-drug challenge in the extracellular concentration of NA (**a**) and volume of distribution V_T_ of [^11^C]yohimbine (**b**). (**a**) Percent change of extracellular NA concentration is shown during the time frame of scans 1 and 2 and scans 1 and 3 for the test–retest (*n* = 3), nisoxetine (*n* = 3) and 10 mg/kg (*n* = 1) and 1–2 mg/kg (*n* = 3) amphetamine conditions. The extracellular NA levels in the cortical and thalamic probes were averaged for representation and match the time frame of the PET scans. (**b**) Averaged percent change from baseline in the volume of distribution V_T_ of [^11^C]yohimbine. Percent change is shown for scans 1 and 2 and scans 1 and 3 for the test–retest (*n* = 3), nisoxetine (*n* = 3) and 10 mg/kg (*n* = 1) and 1–2 mg/kg (*n* = 3) amphetamine conditions in the same animals for which dialysis data were available. The V_T_ in the cortical and thalamic regions was averaged for representation. The shading of the test–retest/nisoxetine and amphetamine bars is different to bring attention to the different timing of the scan start time post-challenge (see legend in the graph).

A dose of 10 mg/kg amphetamine induced a large, rapid increase in extracellular NA in the thalamus and frontal cortex, which remained elevated for at least 5 h after administration (Figure 4c). See Figure 5a for the averaged data during the scan duration as a percentage of change in extracellular NA from baseline, showing a six-fold increase in NA levels in the thalamus and cortex. Unfortunately, the striatal NA data from this animal were used to calibrate and optimise the HPLC for NA measurements and were partly lost due to technical problems. However, the striatal concentration of DA displayed the expected large increase in DA release following amphetamine (Figure 4c). Concurrently, the sustained increase in extrastriatal extracellular NA over several hours induced a sustained 20–25% decrease in [^11^C]yohimbine V_T_ in the thalamus over the same period of time (Figure 5b). The decrease in striatal V_T_ was, however, less pronounced (12%).

Lower doses of 1 or 2 mg/kg amphetamine induced smaller increases in cortical and thalamic extracellular NA, leading to a smaller decrease in [^11^C]yohimbine V_T_ (Figure 4d and Figure 5). The increase in striatal NA was less pronounced than in the cortex and thalamus but there was still a robust increase in striatal DA (see Figure 4d). Similarly, a smaller decrease in striatal V_T_ (10%) was noted compared to other regions (15%).

Because nisoxetine blocks the reuptake of NA but does not increase its release, the concentrations of NA in the cortical and thalamic dialysate increased slowly over a couple of hours following a 1 mg/kg nisoxetine challenge (Figure 4b and Figure 5a). In parallel, [^11^C]yohimbine binding was only minimally decreased (7%) in the first scan starting 5–20 min post-nisoxetine but decreased further in the follow-up scans 2–3 h after administration (13.5%) (Figure 5b). There were minimal changes in extracellular NA and DA (Figure 4b) in the striatum.

**Figure 6:**
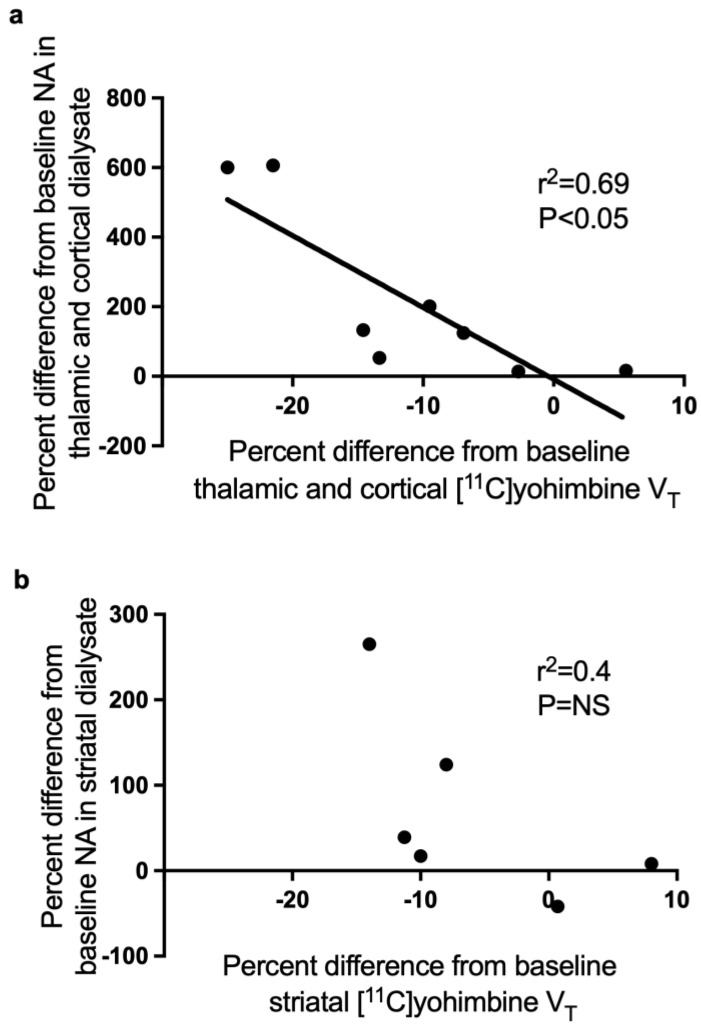
Correlations between the variations in extracellular NA and yohimbine V_T_. Data are expressed as percent changes from baseline in thalamus and cortex (**a**) and in striatum (**b**). Each point represents the average % change in dialysis vs. the average % change in PET for each condition (4 conditions) and each timepoint (2 time comparisons). There was a significant inverse correlation between extracellular NA and V_T_ in regions of high NA innervation and pre-eminence of α2A postsynaptic receptors. This correlation is however absent in the striatum, a region of sparse NA innervation.

There was a significant inverse correlation between extracellular NA and yohimbine binding in the cortex and thalamus (Figure 6, r^2^ = 0.69, *p* < 0.05), and no significant correlation in the striatum (r^2^ = 0.4).

Under test–retest conditions, with inter-scan intervals similar to those used in the nisoxetine challenge studies, the average change in [^11^C]yohimbine V_T_ between scans 1, 2 and 3 was 5–8% (ranging from −4 to +17% depending on the region and the scan). Figure 5b shows the average percent change between scan 1 and 2 and scan 1 and 3 for the test–retest studies. The measured values fall within the previously determined averaged test–retest range for pigs [15]. Consistent with the lack of change in [^11^C]yohimbine binding, no major alterations in microdialysis data for NA or DA were observed throughout the day (see Figure 4a and Figure 5a).

## 4. Discussion

Here, we confirm our earlier preclinical imaging data [15,22,23] suggesting that the binding of [^11^C]yohimbine is sensitive to acute changes in the concentration of extracellular NA using two challenges that exert a temporally different effect on NA using a specialised dual PET-microdialysis setup. While [^11^C]yohimbine appears to be a useful tracer for measuring in vivo acute changes in extracellular NA, several aspects of the study deserve further discussion.

The variability in baseline concentrations of NA between regions and animals (see Figure 4) may reflect physiological differences as well as anaesthesia-related alterations and variability due to external factors such as the placement of the probes and percentage of grey–white matter covered by the dialysis membrane. Thus, while we present in Figure 4 individual data as a visual representation of the effects of challenges, we based our conclusions on the average percent change from baseline following an intervention during the time frame of the scans. To summarise the results, we pooled and averaged the PET and dialysis data for the cortex and thalamus to maximise the number of subjects available for comparison. This decision was based on several observations: specifically, yohimbine does not distinguish between NA α2 receptor subtypes and thus binds equally to the A and B subtypes found primarily in the cortex and thalamus [12,13]. Furthermore, the cortex and thalamus displayed a relatively similar response (see Figure 4) to the individual challenges. Figure 5a shows the averaged responses: rapid, dose-dependent increases in extracellular NA in response to R-amphetamine, a potent releaser of DA and NA, and a slow, progressive increase in response to nisoxetine, a non-releasing inhibitor of the NET with negligible affinity for the DA and serotonin transporters [35]. As expected, amphetamine increased DA in striatum but only moderately affected striatal NA. Similarly, nisoxetine had little effect on extracellular striatal NA or DA (see Figure 4). Indeed, the striatum receives sparse NA innervation compared to almost every other brain region: the caudate nucleus has even been used as a region of non-specific binding to measure the binding potential of PET tracers of the NET [36,37]. Furthermore, the major α2 receptor subtype in the striatum is reportedly α2C receptors (60–70%), which has a similar affinity for DA and NA [31]. DA has even been proposed as the main ligand for the α2C receptors [30]. As the affinity of DA for the α2 receptors is only 1.5 to 3 times less than that of NA (4 to 6 μM) [30], large concentrations of extracellular DA may compete with NA for the α2 binding sites, especially in the striatum, where DA concentrations are the brain’s highest.

Our data need to be considered in view of the report by a Finnish group that labelled ORM-13070 as a selective marker of α2C receptors and validated it for human use [38]. They proposed this tracer to be a surrogate marker of NA neurotransmission based on drug challenges, including ketamine and atomoxetine combined with cold stimulation, known to increase NA levels, which led to reduced striatal ORM-13070 binding [38]. However, in the absence of microdialysis data in the same subjects, they provide only indirect evidence that [^11^C]ORM-13070 is a specific surrogate marker of NA release, especially in the striatum, which receives minimal NA innervation but has massive DA innervation, especially in primates. Like the Finnish investigators, we observed a strong displacement of striatal yohimbine binding in non-human primates administered amphetamine [39]. However, based on the paucity of striatal NA innervation, the pre-eminent distribution of α2C receptors in the striatum and their strong affinity for DA, it remains unclear if the striatal amphetamine-induced changes in ORM-13070 or yohimbine binding reflect changes in NA solely or, more likely, a combined NA-DA stimulation, which we propose is occurring in striatal areas, based on our combined PET and microdialysis data. In support, a recent human study investigated the potential of clonidine, an α2 agonist known to decrease catecholamine release, to increase [^11^C]yohimbine binding, and reported increased binding in the striatum, amygdala and posterior cortical areas [40].

Consideration of the yohimbine PET data merits some further comments. While there were regional differences in V_T_ throughout the brain, in the regions of α2A-B predominance, such as the cortex and thalamus, the percent decrease from baseline was relatively similar. We thus averaged cortical and thalamic regions to more easily compare the dialysis and imaging data. Figure 5b shows that yohimbine binding decreases as extracellular NA increases. Amphetamine induced an immediate and profound dose-dependent decrease in V_T_. Binding following nisoxetine administration, on the contrary, decreased minimally initially but continued to decrease over time as extracellular concentrations increased. Due to the length and invasive nature of these fourteen-hour experiments, we did not collect data at late enough time points in order to ascertain if the binding of the PET tracer returns to baseline within the same time frame as extracellular NA dialysis data.

Despite the low number of animals included in our study, there was a significant inverse correlation between extracellular NA and yohimbine binding in areas of predominant α2A-B receptors (Figure 6). Using only cortical and thalamic nisoxetine data in order to avoid the possible confounding effects of the mix of NA with large DA concentrations on [^11^C]yohimbine binding, we calculated the percentage increase in NA concentrations required to induce a 1% increase in V_T_ to be approximately 16%.

On the contrary, there was no correlation between yohimbine binding and extracellular NA in the striatum, where the majority of α2 receptors belong to the α2C subtype, a subtype of α2 receptors believed to have DA, not NA, as its major ligand [31]. Indeed, there are few NA terminals in the striatum and the caudate nucleus has even been used as an area of non-specific binding for the noradrenaline transporter tracer MRB [36,41]. Because many striatal samples were used to optimise the DA dialysis protocol, we did not have sufficient data to demonstrate if striatal yohimbine binding more purely reflected extracellular DA or a combination of DA and NA, but this hypothesis cannot be excluded.

A further limitation of our study is practical: the location of the catheters was verified in vivo by CT and in vitro by post-mortem histology, and it was apparent that the location was not always ideal. We used human-size catheters to improve the sampling efficiency but the 10 mm length of the dialysis membrane may have contributed to the increased variability due to a greater mix of grey and white matter in the comparatively smaller pig brain, especially in cortical areas. While we verified the location of the probe tips in the striatum, thalamus and within the frontal cortical regions, we did not specifically measure the mix of white/grey matter in cortical probes or the position of the probe in specific thalamic nuclei (in which α2 receptors have a heterogenous distribution [42]); however, since we avoided getting close to the middle sagittal sinus during probe insertion, the thalamic probes cover more of the lateral nuclei. Further studies may benefit from the use of rodent-size dialysis probes with shorter tips. In order to reduce the implant time, we did not use a guide cannula and the dialysis probe itself was guided into the brain while the dura matter was opened for the procedure. This may have led to damage of the tip of some probes, as well as bending of the flexible material, leading to some damage visible upon removal and some slightly off-target location. Using a rigid cannula descended through the first cortical layers or above the intended location may improve targeting and protect the fragile tip in minipig brain capped by a large sinus. For the purpose of a PET–dialysis combined study, placement under high-resolution CT guidance was sufficient to identify large structures such as the thalamus and striatum but may not have been enough for guidance to smaller targets. Performing the study in a PET-MR scanner would have undoubtedly improved our dialysis probe placement, but that technology was not available at the time of our study. However, as a whole, the study provided useful and reliable data within each animal.

A recent human study [40] suggested the use of a white matter reference region, specifically the corpus callosum, to replace invasive blood sampling for a kinetic analysis of [^11^C]yohimbine binding. Although we attempted to validate this approach in our minipig dataset, the relatively small size of the minipig brain together with the suboptimal resolution of the PET/CT scanner confounded the data and we could not delineate reliably in all of the pigs a white matter region in either the corpus callosum or central semiovale that was not subject to a variable amount of partial volume contamination by surrounding grey matter.

We confirmed our initial results using [^11^C]yohimbine as a surrogate marker of changes in extracellular NA, following pharmacological challenge with amphetamine [22,23]. Furthermore, we also detected decreased [^11^C]yohimbine binding in minipig following acute VNS [15]. As this brain stimulation therapy has antidepressant effects, we hypothesised that the decrease in tracer binding was due to stimulation of NA transmission. The current study provides further validation of this hypothesis.

In conclusion, we measured NA dialysate in three different brain regions during drug challenges and [^11^C]yohimbine PET in order to begin to untangle the effects of NA release. Amphetamine and nisoxetine, although with different temporal patterns, increased NA levels in thalamic and cortical regions, regions of sparse DA innervation. However, amphetamine strongly affected DA in the striatum, a nucleus where NA terminals are fewer and where the effect remained less pronounced. This provides a suggestion for a specific NA-effect in NA regions and a mixed NA–DA-effect in striatal regions where DA and NA effects may be confounded by the distribution of terminals, as well as of α2 subtypes with heterogenous affinity for NA and DA. Our studies combining two powerful methods to investigate extracellular neurotransmitter levels and receptor binding suggest [^11^C]yohimbine as a surrogate marker at least in regions of predominant NA innervation, with some caution to be exerted in the striatal regions. These results could hold significant implications for the design of future studies in neurology and psychiatry.

## Data Availability

Data are available from the corresponding author upon reasonable request.
